# Synthesis of N-acetyl-L-cysteine-capped ZnCdSe quantum dots ***via*** hydrothermal method and their characterization

**DOI:** 10.1088/1468-6996/15/5/055001

**Published:** 2014-09-08

**Authors:** Fang Gao, Yuying Liu, Yao Fan, Dan Zhao

**Affiliations:** College of Pharmacy, South-Central University for Nationalities, Wuhan 430074, People’s Republic of China

**Keywords:** ZnCdSe, QDs, NAC, hydrothermal route

## Abstract

Compared with the most studied green-red emitting (530–650 nm) quantum dots (QDs), the preparation of short-wavelength-emitting QDs remains difficult. Besides, one of the representative short-wavelength QDs materials, ZnCdSe, has a shortcoming of high content of toxic cadmium metal. In this paper, we report the synthesis of high-quality water-soluble ZnCdSe QDs via optimized one-step hydrothermal method with a new thiol as ligand, within a short time of 65 min. The emission wavelength of prepared QDs is tunable in the range of 425–540 nm by merely controlling the molar ratio of Cd:Zn or Se:Zn, and the quantum yield reaches 35%. More importantly, the maximum Cd:Zn molar ratio has been reduced to 0.04:1.0, much lower than that reported in the literature (0.5:1.0), resulting in excellent biological compatibility of prepared QDs and thus their promising applications in biological fields. Moreover, the transmission electron microscopy was employed to examine the effect of Cd:Zn ratio on the size of prepared ZnCdSe QDs, which were also characterized by x-ray photoelectron spectroscopy and electron diffraction spectroscopy.

## 1. Introduction

Quantum dots (QDs) have received considerable attention over the last few decades, because of their distinguished roles in fundamental studies and technical applications [1–6]. Especially, compared with quantum well (active material in ZnSe-based materials lasers) [7], QDs possess many obvious advantages, including higher differential gain, lower threshold current density, and higher temperature stability [8]. Unfortunately, the binary systems do not work well for the synthesis of the QDs with emission in the short wavelength spectral region from 420 to 500 nm, which is of technological interest for the preparation of QDs-based blue light-emitting devices and white-light generation [9]. For example, the CdSe QDs can emit fluorescence in the blue-green spectral range, but the fluorescence in the blue spectral region requires small particle size (smaller than 2 nm) for such a wide band gap [10], which would lead to the instability problem [11]. Even though wider band-gap materials such as CdS and ZnSe can also be used to prepare blue-emitting QDs theoretically, their photoluminescence (PL) properties are also not very stable in practice [12]. Recent advances in synthesis have led to the exploration of ternary QDs [9] through adjusting the constituent stoichiometries of alloyed semiconductors [11].

ZnSe-based materials have been regarded as the most promising candidates of light emitting and laser diodes in the blue-green spectral region [13, 14], and ZnSe-based colloidal ternary alloy QDs, such as ZnCdSe [9–12, 15–18], ZnMnSe [19, 20] and ZnCuSe [21], have been widely investigated. However, the emission wavelength of ZnMnSe QDs cannot reach the spectral region below 520 nm, and ZnCuSe QDs, though tunable from 488 to 522 nm, possess relatively low quantum yields (QY). On the other hand, hydrophobic Zn_x_Cd_1 − x_ Se-alloyed QDs have been successfully prepared with high QYs (45–70%) and a widely tunable range of emission wavelength from 440 to 550 nm by Zhong *et al* [12]. Along with the necessity of ZnCdSe QDs in many optoelectronic applications, the realization of laser diodes based on long-lifetime ZnCdSe makes the study of ZnCdSe QDs even more attractive [22].

Recently, aqueous-phased synthesis of QDs with thiols as the capping agent has attracted significant attention for their application in biological imaging [16]. Whilst, at present, the synthesis of ZnCdSe QDs are mainly through organometallic method [9, 11, 12, 15, 17, 23]. Besides, some groups also employed directly aqueous synthesis method [16, 24]. For both reported organometallic method and the directly aqueous synthesis method, there remains one common problem to be solved, that is, the high dose of Cd in prepared QDs, which would greatly prevent their application in biological areas due to the toxicity of Cd. For instance, through organometallic method, the minimum Cd:Zn molar ratio in Zhong’s paper was 0.5:1.0, while the amount of Zn precursor solution was 0.20 mmol [12]. Moreover, through aqueous synthesis, Liu reported the increase of the Cd:Zn molar ratio from 0.03 to 0.2 (the total amount of Zn and Cd was 0.4 mmol) resulted in a red shift of emission peaks from 430 to 505 nm [16]. Reducing the doping ratio of Cd has been the key point and difficulty in the synthesis of ZnCdSe QDs, especially taking their application in biological areas into account.

On the other hand, although studies showed that higher reaction temperature has been contribute to the homogeneous alloyed structure and thus better optical performance, the commonly-used reflux method in aqueous phase was usually operated at the temperature of 80 or 95 °C. As a newly-developed aqueous method, the hydrothermal route can greatly speed up the process initiated by its high reaction temperature (>100 °C), and thus reduces the surface defects and ensures high fluorescence properties of as-prepared QDs [25, 26]. Therefore, it has been regarded as an ideal method for direct synthesis of high-quality water-soluble QDs.

The selection of ligand is also of great importance for the properties of prepared QDs in aqueous phase method. Liu [16] has tested the impact of thiols on the synthesis of Zn_x_Cd_1 − x_Se QDs, including 3-mercaptopropionic acid, thiolglycolic acid, thiolactic acid, glutathione and cysteine, and the results suggested that cysteine provided better surface passivation of the QDs crystalline lattice under the same condition. As a derivative of L-cysteine, *N*-acetyl-L-cysteine (NAC) is known as an antioxidant to protect cells against oxidative stress and QD-induced cytotoxicity [27], and it possesses excellent biocompatibility, water-solubility and user/environmental-friendliness. Our previous researches also showed that NAC is an excellent ligand under the high reaction temperature and pressure in hydrothermal route [25, 26, 28].

Moreover, different from the binary quantum dots, the properties of ternary alloyed QDs can be greatly influenced by the reaction order of three elements, which, however, is often ignored. Since Cd^2+^ is considerably more reactive than Zn^2+^ toward NaHSe [16] due to the much lower bond dissociation energy of Zn-Se (136 kJ mol^−1^) than that of Cd-Se (310 kJ mol^−1^) [11], we propose that NaHSe should firstly react with Zn^2+^ to form ZnSe nuclei before the addition of Cd^2+^. The cation exchange reaction could happen between ZnSe seeds and Cd^2+^, and is beneficial to form colloidal ternary alloy ZnCdSe QDs. The synthesis environment is another factor that greatly influences the properties of prepared QDs, and the impacts of various experimental parameters (*viz*. pH, reaction time, and molar ratio of reactants) thus need to be systematically investigated.

In this paper, we report the use of NAC as the stabilizer to synthesize a series of high-quality ZnCdSe QDs via one-pot hydrothermal route by incorporating a Cd precursor into the newly formed ZnSe nuclei together with the remaining Zn/Se precursors. The as-prepared NAC-capped ZnCdSe QDs exhibit excellent water solubility, stability and excellent QYs. This method greatly reduced Cd content in prepared QDs, and also realized simple, wide-range fluorescence tuning (420–496 nm) through controlling the Cd:Zn molar ratio (0.005 to 0.04) or Se:Zn molar ratio (0.03 to 0.15). The optimum reaction condition has been found out via a series of optimizing experiments. Furthermore, we characterized the optical properties of obtained ZnCdSe QDs through various methods. Transmission electron microscopy (TEM) was employed to study the particle diameter and distribution of the prepared QDs with different n(Cd)/1(Zn) values. Finally, we realized qualitative and semi-quantitative research on the components of prepared ZnCdSe QDs. NAC-capped ZnCdSe QDs not only meet the current requirements for fluorescence materials in light-emitting devices and lasers, and are sure to have extensive and promising applications in future biomedical fields.

## 2. Experimental procedures

### 2.1. Chemicals

Selenium (reagent powder) was purchased from Shanghai Mei Xing Chemical Co. Ltd CdCl_2_, ZnCl_2_, quinoline sulfate and sodium borohydride (NaBH_4_) were obtained from Sinopharm Chemical Reagent. NAC was purchased from Sigma. All chemicals used were of analytical grade or of the highest purity available. All solutions were prepared using Milli-Q water (Millipore) as the solvent.

### 2.2. Preparation of ZnCdSe QDs

Sodium borohydride was used to react with selenium powder in deionized water (3.0 mL) to produce sodium hydroselenide (NaHSe, 0.0844 mol L^−1^). Fresh NaHSe solutions were then kept into freezer for further use. ZnCl_2_ (6.4 mmol L^−1^) and a certain amount of NAC were dissolved in 250 mL of deionized water and stirred vigorously for 20 min. The precursor solution was adjusted to desired value (8 to 11) by stepwise addition of 1.0 mol L^−1^ of NaOH. Subsequently, the fresh NaHSe solution (0.0844 mol L^−1^) was added to a N_2_-saturated mixture of ZnCl_2_ and NAC by a certain percentage and stirred vigorously for 5 min. Then, a certain amount of CdCl_2_ (0.05 mol L^−1^) were injected into the above prepared solution and stirred vigorously for 5 min more. The Zn^2+^ concentration was 6.4 mmol L^−1^ in a total volume of 50 mL. The molar ratios of NAC/Zn^2+^ used in our experiment are 1.2:1, 2.0:1, 2.4:1, 3.0:1, and 3.6:1, in sequence. The molar ratios of HSe^−^/Zn^2+^ used in our experiment are 0.03:1, 0.04:1, 0.05:1, 0.07:1, 0.10:1, and 0.15:1, in sequence. The molar ratios of Cd^2+^/Zn^2+^ used in our experiment are 0.005:1, 0.01:1, 0.02:1, 0.03:1, and 0.04:1, in sequence. Finally, the resulting mixture was put into a 50 mL Teflon-lined stainless steel autoclave. It was loaded in an oven at 200 °C for a specified time (50 to 70 min) and then cooled to the room temperature by a hydrocooling process.

To remove NAC-Zn/Se complexes at the end of the synthesis, cold 2-propanol was added to the reaction mixture to precipitate NAC-capped ZnCdSe QDs. The as-prepared products were dried overnight under vacuum at 40 °C for further experiments.

### 2.3. Cell culture and *in vitro* cytotoxicity assay of the NAC-capped ZnCdSe QDs

L6 myoblast cells were cultured in a humidified atmosphere containing 5% CO_2_ at 37 °C using *α*-modified eagle medium (*α*-MEM) supplemented with 10% heat-inactivated fetal bovine serum (FBS), 100 U mL^−1^ of penicillin G, and 100 *μ*g mL^−1^ of streptomycin. The cytotoxicity of the QDs-based samples was accomplished employing the colorimetric 3-(4,5-dimethylthiazol-2-yl)-2,5-diphenyltetrazolium bromide (MTT) technique. Briefly, the cells were grown in 96-well plates at a density of 1.3 × 10^5^ cells/mL 12 h prior to the exposure to 1 *μ*M QDs-based samples for 24 h. Afterward, cells were washed and treated with MTT solution (0.5 mg mL^−1^, final concentration) for 4 h. Finally, the supernatant was removed, and dimethyl sulfoxide (DMSO) (150 *μ*L) was added to solubilize the formed formazan salt. The amount of the formazan salt was determined by measuring the absorbance at 570 nm using a microplate reader (TECAN Infinite M200 PRO, Switzerland). The cell cytotoxic level was quantified as a percentage compared to the blank (without addition of QDs). The cell viability was expressed as: Cell viability = (OD_x_ − OD_o_)/(OD_s_ − OD_o_) × 100%, where OD_x_, OD_o_ and OD_s_ represent the absorption of test samples, blank control ones and negative control ones at 570 nm, respectively.

### 2.4. Characterization

UV-visible absorption spectra were acquired with a Lambda-35 UV/visible spectrophotometer (PerkinElmer Company) to determine the bandgap absorption of QDs. Fluorescence spectra were recorded on a LS55 spectrofluorometer (PerkinElmer Company). All optical measurements were performed under ambient conditions. The TEM sample was prepared by dropping an aqueous ZnCdSe QDs solution onto an Agar carbon-coated copper grid (400 meshes) with the excess solvent evaporated. The TEM image was obtained at 310 K magnification with an FEI Tecnai G220 twin transmission electron microscope. X-ray diffraction (XRD) patterns were recorded on a Shimadzu XRD-2000 x-ray diffractometer. Energy-dispersive x-ray spectroscopy (EDS) measurements were performed with an FEI Quanta 200 scanning electron microscope equipped with an energy dispersive x-ray spectrometer. X-ray photoelectron spectroscopy (XPS) measurements were carried out with a Leybold Heraeus SKL 12 x-ray photoelectron spectrometer. The QY of ZnCdSe QDs was measured according to the literature [29]. Quinoline sulfate in 1 mol L^−1^ H_2_SO_4_ aqueous solution was chosen as the reference standard (QY = 54.6%).

## 3. Results and discussion

### 3.1. Synthesis of NAC-capped ZnCdSe QDs.

#### 3.1.1. Influence of the pH of precursor solution

**Figure 1.**
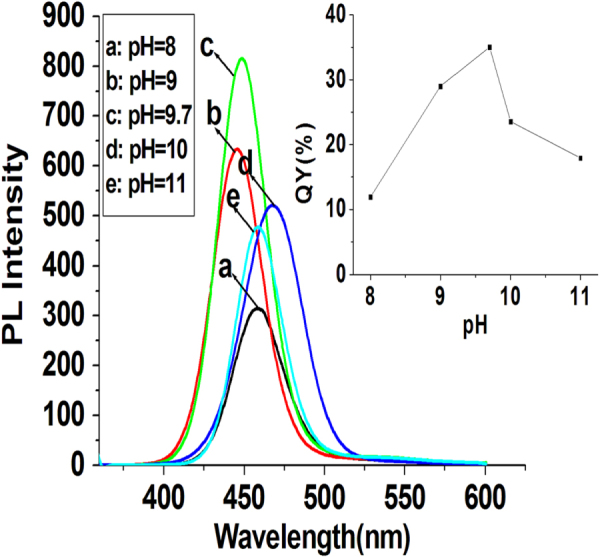
PL spectra of NAC-capped ZnCdSe QDs grown at different pH values (*λ*_ex_ = 310 nm); the inset shows the QYs of these prepared QDs.

The pH environment during the synthesis can greatly influence the fluorescence properties of the prepared QDs. The precursor solutions were operated in alkaline conditions (pH 8–11). Further lowering the pH would lead to luminescence decrease or precipitation of the ZnCdSe QDs, possibly arising from the aggregation of the QDs [26]. As shown in figure 1, with other reaction factors fixed ([Zn^2+^] = 6.4 mmol L^−1^, molar ratio of Zn:Cd:Se:NAC at 1.0:0.02:0.1:3.0, reaction temperature at 200 °C, and reaction time at 65 min), the best QY of prepared QDs occurs at pH = 9.7, suggesting that the weakly alkaline reaction environment is beneficial to the formation of high-quality QDs. In contrast, the pH value did not have obvious influence on the emission peaks of prepared QDs, which maintained near 456 nm with pH increasing from 8 to 11.

#### 3.1.2. Influence of reaction time

**Figure 2.**
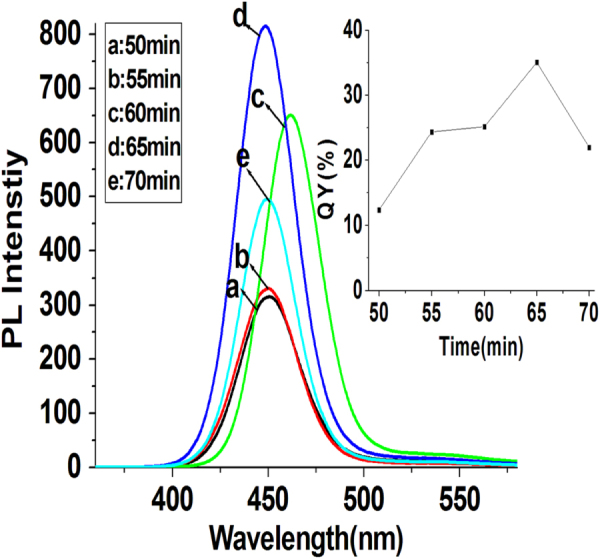
PL spectra of NAC-capped ZnCdSe QDs prepared at various reaction times (50 to 70 min) (*λ*_ex_ = 310 nm); the inset shows the QYs of these prepared QDs.

We discovered that the reaction time of QDs synthesis also played an important role in controlling the QY of as-prepared ZnCdSe QDs. With other reaction factors ([Zn^2+^] = 6.4 mmol L^−1^, molar ratio of Zn:Cd:Se:NAC at 1.0:0.02:0.1:3.0, pH = 9.7, and reaction temperature at 200 °C) fixed, we gradually changed the reaction time (50–70 min) to testify the impact of reaction time upon the prepared QDs. As shown in figure 2, the single peak of fluorescence emission rules out separate nucleation and growth of CdSe and ZnSe [15, 17]. Because appropriate amount of NaHSe was added into the Zn^2+^-NAC solution five minutes earlier than the injection of Cd^2+^, the synthesis of ZnCdSe QDs started from the formation of ZnSe seeds. Since the bonding dissociation energy of Zn-Se (136 kJ mol^−1^) is relatively much lower than that of Cd-Se (310 kJ mol^−1^) [11], Cd^2+^ is considerably more reactive toward NaHSe than Zn^2+^ does [16], leading to the cation exchange reaction between ZnSe seeds and Cd^2+^ [11]. We regard that the prepared QDs are in the form of ZnCdSe alloyed structure covered by ZnSe shell because the trace amount of Cd^2+^ in the system (Cd^2+^/Zn^2+^ = 0.02:1) would be quickly consumed up within very short time before the formation of ZnSe shell. It’s clear that the reaction time does not significantly affect the emission peaks (452 nm) and absorption peaks (419 nm), but affects the QY of the prepared ZnCdSe QDs obviously. The shorter reaction time leads to more surface defects and lower QY. On the contrary, though the longer reaction time is beneficial to the synthesis of high-quality QDs, when the reaction time is longer than 65 min, the decomposition of NAC will deprive its ability as a stabilizer, and thus cannot produce QDs with good properties [28].

#### 3.1.3. Influence of the molar ratio of NAC to Zn

**Figure 3.**
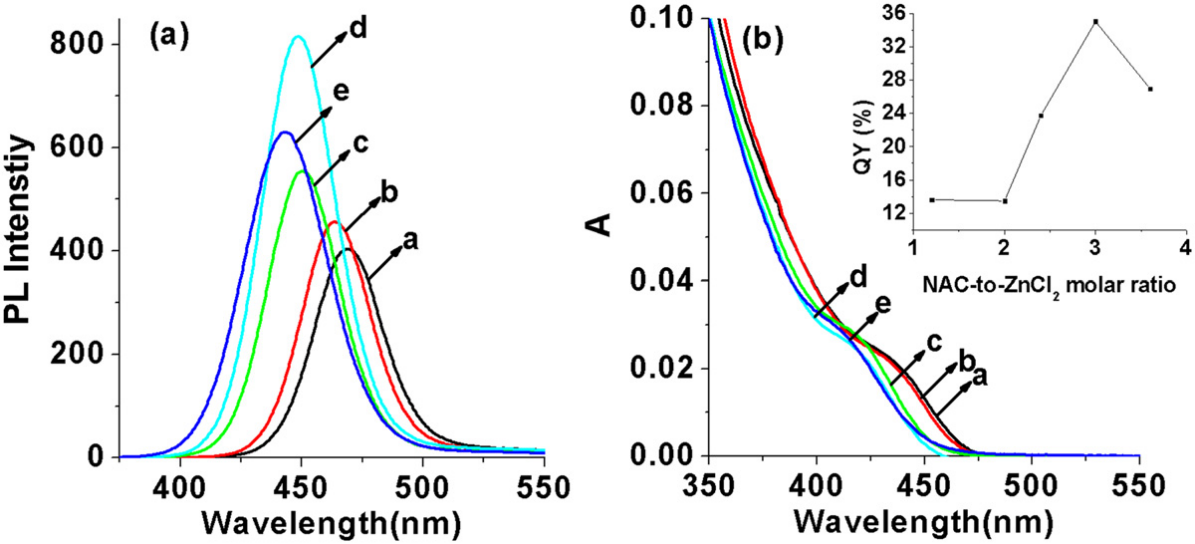
(a) PL spectra and (b) UV-visible absorption spectra of NAC-capped ZnCdSe QDs with different n(NAC)/1(Zn) values (1.2, 2.0, 2.4, 3.0, and 3.6 from a to e) (*λ*_ex_ = 310 nm); the inset shows the QYs of these prepared QDs.

An excellent ligand NAC was employed as the capping reagent here, which could not only serve as the stabilizer to ensure the water-solubility of prepared QDs, but also passivate the surface of prepared QDs very well in the synthesis to decrease the surface defects. Consequently, the concentration of NAC is an important factor that affects the luminescent properties of ZnCdSe QDs. By changing the addition amount of NAC with other reaction parameters ([Zn^2+^] = 6.4 mmol L^−1^, molar ratio of Zn:Cd:Se at 1.0:0.02:0.1, reaction time at 65 min, pH = 9.7, and reaction temperature at 200 °C) fixed, the QY reached the highest when the Zn:NAC ratio is 1.0:3.0 (figure 3). Meanwhile, when the ratio increased from 1.2 to 3.6, the emission peaks of prepared QDs shifted from 469 to 443 nm and the absorption peaks (*λ*_max_) moved from 434 to 409 nm, indicating that the increasing amount of NAC would stick to the surface of crystal nucleus or bond to the Zn precursors through the thiol group, slowing down the growth rate of particles [10] and thus reducing their particle diameters.

#### 3.1.4. Influence of the molar ratio of constituent elements

**Figure 4.**
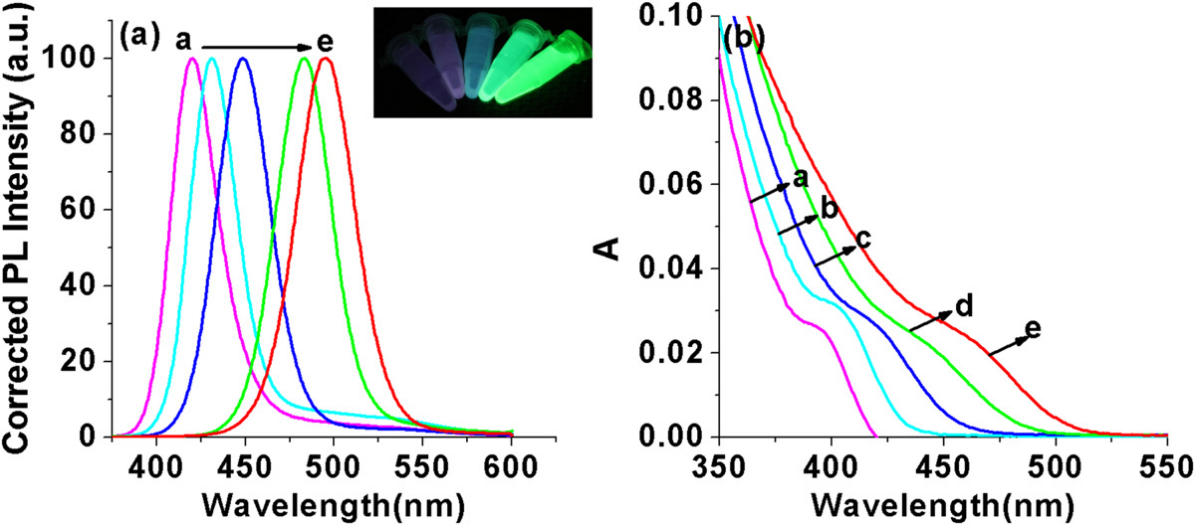
(a) Corrected PL spectra and (b) UV-visible absorption spectra of NAC-capped ZnCdSe QDs with different n(Cd)/1(Zn) values (0.005, 0.01, 0.02, 0.03, and 0.04 from a to e) (*λ*_ex_ = 310 nm); the inset shows the PL image of the corresponding ZnCdSe QDs under UV lamp irradiation.

The composition of the ZnCdSe QDs mainly depends on the amount of Zn^2+^ and Cd^2+^ precursors and the intrinsic Zn^2+^ and Cd^2+^ reactivities toward NaHSe. Adjustment of n(Cd)/1(Zn) values provides a simple way for preparing color-tunable ZnCdSe QDs [16]. Consequently, we fixed the Zn:Se:NAC molar ratio (1.0:0.1:3.0) and changed the addition amount of Cd^2+^. As stated previously, since Cd^2+^ is more active to interact with NaHSe than Zn^2+^, we firstly added NaHSe into Zn precursor solution, and then added Cd^2+^. The dose of Cd used in our system is much less than that reported in published literatures [12, 15, 16]. As shown in figure 4, with other reaction parameters fixed (pH = 9.7, reaction time at 65 min, reaction temperature at 200 °C, and Zn concentration at 6.4 mmol L^−1^), the increase of the Cd:Zn molar ratio from 0.005 to 0.04 resulted in a red shift of emission peaks from 420 to 496 nm and of absorption peaks (*λ*_max_) from 396 to 459 nm. The red-shift (figure A.1(a), see supplementary data available from stacks.iop.org/STAM/15/055001/mmedia) was caused by the first excitonic absorption wavelength decreasing with the incorporation of the narrower band-gap CdSe (e.g. = 1.74 eV) into the starting wider band-gap ZnSe (e.g. = 2.62 eV) [12]. The emission color under UV irradiation turned from purple to bluish violet and then blue-green with the increase of Cd: Zn molar ratio (the inset of figure 4). Meanwhile, the average QY was 26% and reached the highest (35%) when the Cd:Zn ratio was 0.02:1.0.

**Figure 5.**
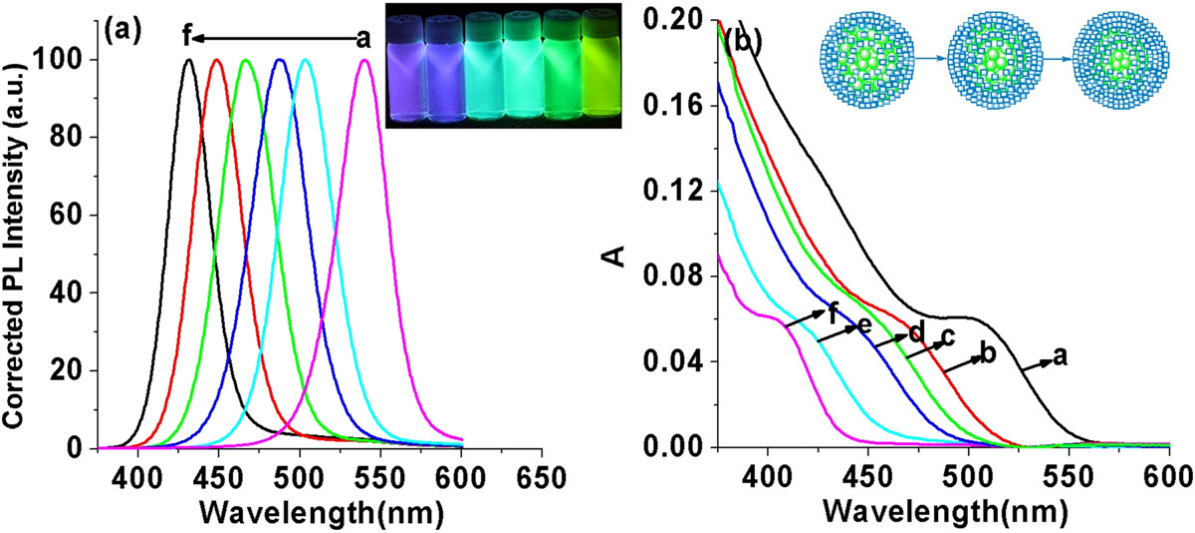
(a) Corrected PL spectra and (b) UV-visible absorption spectra of NAC-capped ZnCdSe QDs with different n(Se)/1(Zn) values (0.03, 0.0 4, 0.05, 0.07, 0.10, and 0.15 from a to f) (*λ*_ex_ = 310 nm); the left and the right insets show the PL image of the corresponding ZnCdSe QDs under UV lamp irradiation and the schematic representation of spherical QDs with increasing n(Se)/1(Zn) values respectively.

To understand the impact of the NaHSe concentration upon the system, the molar ratio of Zn:Cd:NAC was fixed at 1.0:0.02:3.0, and gradually increased the Se:Zn ratio from 0.03 to 0.15. Since the concentration of Cd^2+^ is much lower than that of Zn^2+^, in order to realize full reaction of Cd^2+^, the addition amount of Se should be higher than the dose of Cd^2+^ precursor. As shown in figure 5, the enhancement of the Se:Zn ratio leads to an obvious blue shift of emission peaks from 540 to 425 nm and of absorption peaks (*λ*_max_) from 505 to 401 nm. We assumed that when the dose of NaHSe was at low level, the formation of CdSe (e.g. = 1.74 eV) is the dominant reaction, and only a small amount of NaHSe reacts with Zn^2+^ due to lower reactivity of Zn^2+^ toward NaHSe than that of Cd^2+^ [16, 30]. With the increase of NaHSe, the formation of more ZnSe (e.g. = 2.62 eV) led to the blue-shift in wavelength with the band-gap increased.

As shown in figure A.1(b), at the initial stage, the little enhancement of the molar ratio of Se from 0.03 to 0.04 leads to an obvious blue shift of emission peaks from 540 to 503 nm. However, with further increase of Se, the emission peaks does not have such obvious shift. It suggests that the main constitution of obtained QDs has already been ZnSe. Consequently, it is of no significant meaning to further increase the amount of Se. The emission color under UV irradiation turned from purple to blue, and finally yellow-green with the increase of Se:Zn molar ratio (the left inset of figure 5). Meanwhile, the average QY was 24% and reached the highest when the Se:Zn ratio is 0.1:1.0. The right inset of figure 5 schematically shows the difference between the structures of QDs with different Se:Zn ratio. The composition of ZnSe in outer parts of the QDs increased with the increase of Se:Zn ratio, which is different from the core–shell structure, where a thin layer of a wider band gap semiconductor is grown on the surface of a core semiconductor [11]. Therefore, the trace amount of toxic heavy metal Cd mainly existing in the internal structure greatly improves the biological security and biological compatibility of prepared QDs.

### 3.2. Physicochemical properties of NAC-capped ZnCdSe QDs

Figure A.2(a) displays the pH effect on the emission intensity of NAC-capped ZnCdSe QDs (*λ*_em_ = 449 nm). NAC-capped ZnCdSe QDs exhibit better stability in neutral or alkaline solution but the intensity drops dramatically at lower pH. This is the result of the protonation of the thiol moiety of prepared ZnCdSe QDs under acidic conditions, leading to the detachment of the capping agent from QDs. As such, the QD-ligand complexes are destroyed and consequently decrease their PL intensity. The carboxylic acid moiety of NAC is deprotonated in neutral and basic aqueous solutions. Especially under high pH conditions, the negative charges of carboxylate groups located on the surface of the QDs repel each other, preventing aggregation of prepared QDs, and are thus beneficial to the stabilization of QDs and the occurrence of higher PL efficiency [28]. The fluorescence of NAC-capped ZnCdSe QDs is relatively stable around pH 7 and remains increasing up to pH 11.5, indicating that it can preserve their initial optical properties in physiological environment.

Since QDs are widely used for probing and locating signal transfer-related molecules in cell studies, their degree of biocompatibility, to some extent, determines their biological and biomedical applications. It is thus of great importance in reducing the toxicity and essentially preserving their initial optical properties in physiological environment. The toxicity of studied materials *in vitro* and *in vivo* are affected by multiple factors [31], including materials combinations, size ranges, and surface capping materials, and for Cd-containing QDs, the Cd content, or the release of Cd ions, is the important factor that affect the biocompatibility of prepared QDs [32]. On this point, we have tested the biocompatibility of the prepared QDs by employing MTT technique. To our delight, cytotoxicity results showed that the viabilities of L6 myoblast were observed to be 85.8% when the concentration of NAC-capped ZnCdSe QDs reached 1 *μ*M, suggesting that NAC-capped ZnCdSe QDs may be suitable for biomedical applications.

To examine the photo-stability of the prepared QDs, the ZnCdSe QDs (*λ*_em_ = 449 nm) at an extremely low concentration (abs = 0.01) in Na_2_CO_3_-NaHCO_3_ buffer (0.05 mol L^−1^, pH = 10) were irradiated by a xenon lamp (16 W). As shown in figure A.2(b), the fluorescence decreased by only 6% within 120 min, revealing excellent photostability of the prepared QDs. This is attributed to the excellent ligand NAC, which can passivate the surface of ZnCdSe QDs well and reduce the surface defects.

### 3.3. The characterization of NAC-capped ZnCdSe QDs

#### 3.3.1. TEM and XRD

**Figure 6.**
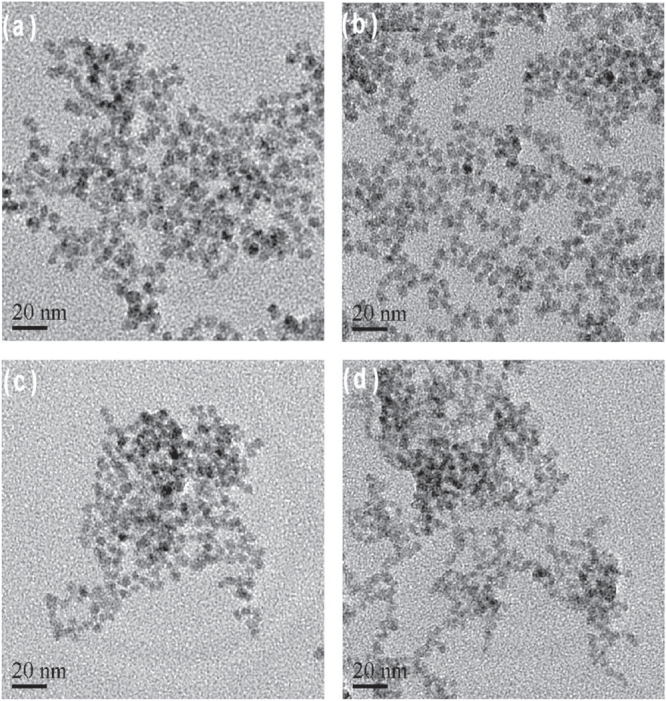
TEM images of a series of NAC-capped ZnCdSe QDs grown at different n(Cd)/1(Zn) values (from (a) to (d): 0.005, 0.02, 0.03 and 0.04). All scale bars are 20 nm.

**Figure 7.**
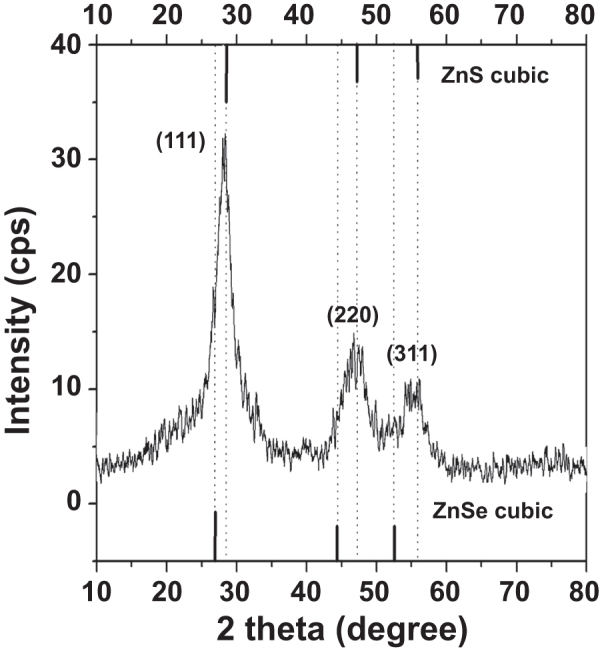
XRD pattern of NAC-capped ZnCdSe QDs reacted for 65 min.

The morphology of the as-prepared NAC-capped ZnCdSe QDs with different n(Cd)/1(Zn) values of 0.005, 0.02, 0.03 and 0.04 was studied by TEM. These QDs have a nearly spherical shape, good dispersion, and a uniform diameter of 6.2 ± 0.2 nm (figure 6). These results indicate that the varying value of n(Cd)/1(Zn) has only limited effect on the particle size of ZnCdSe QDs [10]. Consequently, it is certain that the movement of emission peak position from 420 nm to 496 nm in figure 4 is mainly induced by the variation of composition. The as-prepared ZnCdSe QDs with tunable emission wavelength and similar particle size enjoy great advantage in their application in nuclear targeting of living cells. Because the size of the nuclear pores is a dominant factor for the QDs entry, in this case, the trafficking efficiency depends on the size of the peptide-QDs conjugates [33]. The powder XRD patterns of NAC-capped ZnCdSe QDs (*λ*_em_ = 449 nm) is shown in figure 7. The three strong peaks with 2*θ* values of 28.19, 46.84, and 55.00 degrees correspond to the (111), (220), and (311) planes respectively. The diffraction patterns of zincblende ZnCdSe QDs is intermediate between those of zincblende ZnSe and ZnS materials. This fact can be attributed to the high temperature during the synthesis that caused a partial decomposition of NAC. The released sulfide ions then reacted with zinc ions to form ZnS.

#### 3.3.2. EDS

**Figure 8.**
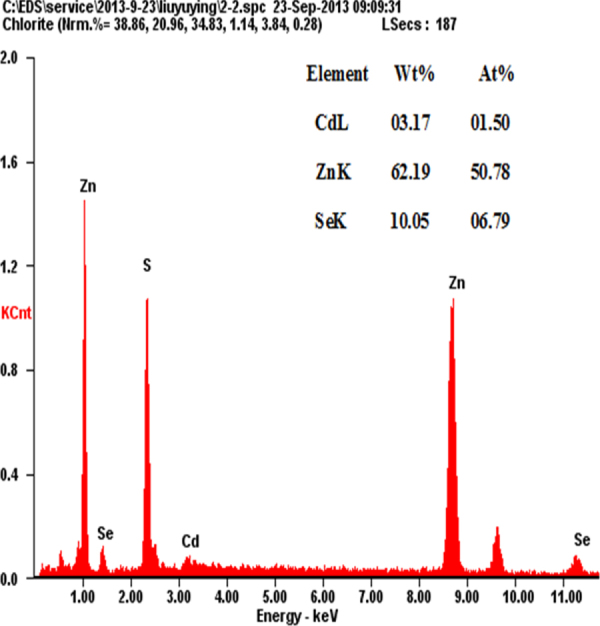
EDS spectrum of NAC-capped ZnCdSe QDs at the molar ratio of Zn:Se:Cd = 1.0:0.1:0.02.

The EDS technique has been employed to estimate the composition of the NAC-capped ZnCdSe QDs. We must emphasize that, for the nano-scaled QDs, the elemental composition obtained through EDS (for which the penetration depth of the electron beam is more than 100 nm) must be the bulk composition of multilayered samples of QDs [26]. As shown in figure 8, for prepared NAC-capped ZnCdSe QDs with molar ratio of Zn:Se:Cd = 1:0.1:0.02 (*λ*_em_ = 449 nm), the atomic percentages of Zn, Se and Cd of the QDs reach 50.78%, 6.79% and 1.50% respectively. The calculated constituent ratio of Zn to Se and Cd is therefore 1:0.13:0.03. While, for another sample of NAC-capped ZnCdSe QDs with molar ratio of Zn:Se:Cd = 1:0.1:0.03 (*λ*_em_ = 483 nm), the atomic percentages of Zn, Se and Cd are 49.98%, 6.92% and 3.12% with the elements content ratio at 1:0.14:0.06 (figure A.3). These results illustrate that the composition of prepared QDs changed with the feed ratio. Compositional EDS data for ZnCdSe QDs are summarized in table A1.

#### 3.3.3. XPS

**Figure 9.**
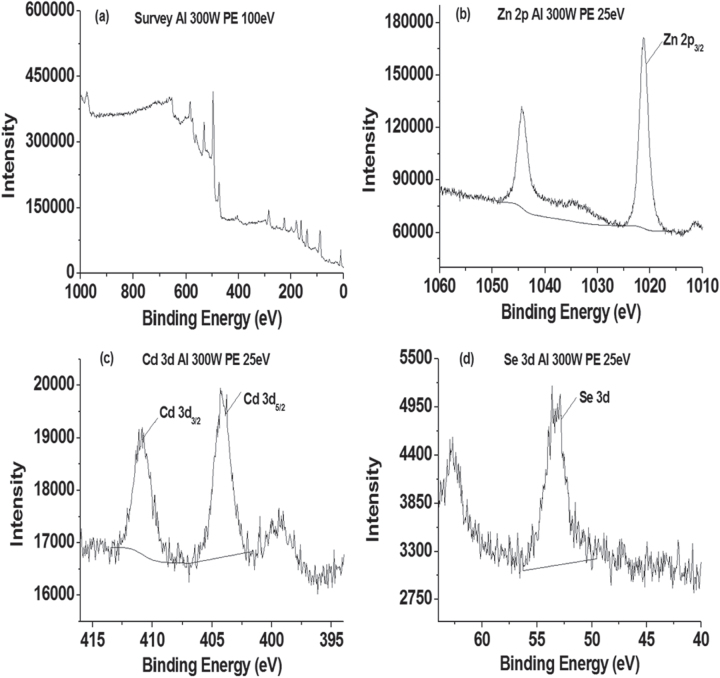
XPS spectra recorded from ZnCdSe QDs (*λ*_em_ = 449 nm): (a) Overview spectrum, (b) Zn (2p), (c) Cd (3d) and (d) Se (3d) peaks.

XPS is a quantitative surface analysis tool, which is sensitive to the atomic composition of the outermost 100 Å of the sample surface [34]. As shown in figure 9 and table A2, the Se (3d) peak at a binding energy of 53.3 eV was assigned to Se bonded to either Zn or Cd [35]. The appearance of characteristic Zn (2p) peaks at a binding energy of 1021.2 eV, which was assigned to the Zn (2p_3/2_) state in ZnCdSe. Similarly, in the Cd (3d) region there were peaks at binding energies of 404.1 and 410.9 eV, which were assigned to the Cd (3d_5/2_) and (3d_3/2_) spin–orbit split states. The results are in accordance with literature values for ZnCdSe [35], illustrating the combination of Zn, Cd and Se. In addition, the Zn:Se:Cd ratio (table A2) deduced from XPS measurements corrected for elemental sensitivity is 1:0.097:0.024 for NAC-capped ZnCdSe QDs (*λ*_em_ = 449 nm), close to the molar ratio of 1:0.1:0.02 of the starting mixture. Furthermore, the results that Se (3d) peak appears at a binding energy of 53.5 eV, Zn (2p) peaks at a binding energy of 1021.4 eV, and Cd (3d) peaks at binding energies of 404.5 and 411.2 eV of another sample of NAC-capped ZnCdSe QDs with molar ratio of Zn:Se:Cd = 1:0.1:0.03 (*λ*_em_ = 483 nm) is also similar to the XPS result of previous sample (figure A.4).

## 4. Conclusions

With NAC as the stabilizer, high-quality water-soluble NAC-capped ZnCdSe QDs with tunable emission wavelengths from 425 to 540 nm have been synthesized through hydrothermal route. The prepared blue-green luminescent ZnCdSe QDs exhibit high QYs and narrow PL spectra. Our results demonstrate that their emission wavelength is tunable by changing the Cd:Zn molar ratio and Se:Zn molar ratio. The luminescence spectra of the obtained QDs show a red-shift (420 to 496 nm) with the increase of Cd:Zn molar ratio (0.005 to 0.04) and a blue-shift (540 to 425 nm) when Se:Zn molar ratio increases (0.03 to 0.15). The luminescence can easily cover the blue-green spectral range through mainly controlling the composition besides controlling the size. Through a series of optimizing experiments, the optimal reaction conditions are found out: molar ratio of Zn:Cd:Se:NAC is 1.0:0.02:0.1:3, pH is 9.7, reaction time is 65 min, and reaction temperature is 200 °C. The morphology of obtained ZnCdSe QDs with different n(Cd)/1(Zn) values were studied by TEM. The results indicate that changing the value of n(Cd)/1(Zn) leads to the variation of composition of the obtained QDs without significant change in particle size. Moreover, we examined the feed ratio and the actual constituent ratio of the prepared QDs through EDS.
